# Lipid production from corn stover by the oleaginous yeast *Cryptococcus curvatus*

**DOI:** 10.1186/s13068-014-0158-y

**Published:** 2014-10-23

**Authors:** Zhiwei Gong, Hongwei Shen, Xiaobing Yang, Qian Wang, Haibo Xie, Zongbao K Zhao

**Affiliations:** Dalian National Laboratory for Clean Energy, Dalian Institute of Chemical Physics, CAS, 457 Zhongshan Road, Dalian, 116023 China; College of Chemical Engineering and Technology, Wuhan University of Science and Technology, 947 Heping Road, Wuhan, 430081 China; Division of Biotechnology, Dalian Institute of Chemical Physics, CAS, 457 Zhongshan Road, Dalian, 116023 PR China

**Keywords:** Biodiesel, *Cryptococcus curvatus*, Cellulase, Xylanase, Corn stover, Microbial lipids, Simultaneous saccharification and enhanced lipid production

## Abstract

**Background:**

Microbial lipids produced from lignocellulosic biomass hold great promise for the biodiesel industry. These lipids usually consist of three major processes: pretreatment, enzymatic hydrolysis and lipid production. However, the conventional strategy of using biomass hydrolysates as the feedstock for lipid production suffers from low lipid coefficient and prohibitively high costs. More cost-effective and integrated processes are required to advance lignocellulosic biomass-based microbial lipid technology.

**Results:**

Three different strategies were tested using the oleaginous yeast *Cryptococcus curvatus* ATCC 20509 as a lipid producer and alkaline-pretreated corn stover as a model material. It was found that the separate hydrolysis and enhanced lipid production process required more cellulolytic enzymes yet afforded a low lipid coefficient of 115.6 mg/g pretreated corn stover. When biomass hydrolysis and lipid production were integrated, the amounts of cellulase and xylanase were reduced and no β-glucosidase was required. The simultaneous saccharification and lipid production process gave a lipid coefficient of 129.4 mg/g pretreated corn stover. A higher lipid coefficient of 159.4 mg/g pretreated corn stover was obtained using the simultaneous saccharification and enhanced lipid production (SSELP) process. Furthermore, cellulolytic enzymes were found recoverable and reusable upon recycling the spent supernatants of the SSELP process, which could reduce enzyme consumption and wastewater discharge.

**Conclusions:**

The SSELP process was superior to other processes in terms of converting alkaline-pretreated corn stover into lipids by *C. curvatus*, as it required less cellulolytic enzymes and had a higher lipid coefficient. Moreover, the process facilitated easy enzyme recycling that should lead to further reduction of enzyme consumption. These results provide valuable information for cost-effective lipid production from lignocelluloses, which should be particularly important in achieving a sustainable production of biodiesel.

## Introduction

Lignocellulose-derived microbial lipids are considered as a potential alternative to vegetable oil as a solution to the feedstock shortage problem that prevents the large-scale production of biodiesel [1]. Three major processes are generally required in order to make lipids from lignocellulosic biomass, namely pretreatment, hydrolysis and lipid production [2,3]. The hydrolysis process uses various enzymes to produce synergistic effects [4]. Although the cost of enzymes has been continuously dropping due to improved specific enzymatic activities, enhanced stability and increased production efficiency, it remains a major obstacle to reaching that required for viable commercial production of biofuels and commodity chemicals from lignocellulosic biomass.

Pretreatment technologies which break biomass recalcitrance can reduce the amounts of enzymes for hydrolysis [5]. In another aspect, enzyme recycling has been widely investigated in biomass hydrolysis studies. Cellulases can either remain bound to the solid residues or be free in the supernatant after the hydrolysis process. Free enzymes in the aqueous fraction are recovered by re-absorption with fresh substrates or by an ultrafiltration procedure [6,7]. Bound enzymes are recyclable by direct contact with fresh substrates [8] or by desorption into the aqueous phase through various methods such as pH adjustment and surfactant treatment [9,10]. When biomass hydrolysis, cell growth and product formation were integrated, fewer cellulolytic enzymes and less time was required [11]. Furthermore, when the production hosts were able to produce cellulolytic enzymes such as β-glucosidase, the amounts of exogenous enzymes required could be further reduced [12,13]. Nonetheless, there were fewer examples on enzyme recovery when integrated processes were involved for the conversion of cellulosic biomass [14].

Microbial lipid production by oleaginous yeasts has been demonstrated using biomass hydrolysates as the feedstock [15-17], but has suffered from low lipid coefficient and prohibitively high costs. To integrate biomass hydrolysis and lipid production processes and enhance the overall efficiency, different processes, namely, separated hydrolysis and enhanced lipid production (SHELP), simultaneous saccharification and lipid production (SSLP) and simultaneous saccharification and enhanced lipid production (SSELP) may be explored. In a recent study, ionic liquid-pretreated corn stover was converted into lipids by the oleaginous yeast *Cryptococcus curvatus* according to the SSELP process, and the lipid coefficient was 112 mg/g regenerated corn stover in the presence of adequate amounts of cellulase, xylanase and β-glucosidase [18]. More recently, we found that *C. curvatus* had the unique feature of converting oligomeric sugars of biomass origin into lipids in the absence of exogenous cellulolytic enzymes, and that oligomeric sugars were transported into *C. curvatus* cells and then hydrolyzed by cytoplasmic enzymes [13].

To further advance microbial lipid technology, three different strategies were tested in this study, using *C. curvatus* as the lipid producer and alkaline-pretreated corn stover as the model material. Special emphases were put on reducing enzyme loading as well as enabling enzyme recovery. Attempts were also made to use recycled enzymes from the SSELP process. Our results provide valuable information for cost-effective lipid production from lignocelluloses, which should be of particular importance in achieving a sustainable production of biodiesel.

## Materials and methods

### Strain, enzymes and materials

*C. curvatus* ATCC 20509 was purchased from the American Type Culture Collection (Manassas, USA), stored at 4°C and propagated every two weeks on yeast peptone dextrose (YPD) agar slants (yeast extract 10 g/L, peptone 10 g/L, glucose 20 g/L, agar 15 g/L, pH 6.0). Yeast extract and peptone were obtained from Aoboxing Biotech. Co. Ltd. (Beijing, China). Yeast inoculum was prepared in the YPD liquid medium (yeast extract 10 g/L, peptone 10 g/L, glucose 20 g/L, pH 6.0).

Cellulase from the fungus *Trichoderma reesei* was purchased from Zesheng Ltd. (Shandong, China). The filter paper activity and cellobiase activity of the cellulase sample were 175.3 filter paper unit (FPU)/mL and 26.1 cellobiose unit (CBU)/mL, respectively [19,20]. β-Glucosidase from *Aspergillus niger* was purchased from Sigma (St. Louis, USA) and had an activity of 674.7 CBU/mL. Xylanase, with an activity of 33.3 kU/g, was obtained from Imperial Jade Bio-Technology Co., Ltd. (Yinchuan, China). One unit of xylanase (U) was defined as the amount of enzyme which produces 1.0 μM xylose from 1% xylan solution within 1 minute at pH 5.0 and 50°C.

Corn stover harvested from the countryside of Changchun, China, was milled and passed through an 18 mesh screen (1 mm size screen). The milled materials were washed to remove field dirt, dried at 105°C to a constant weight, and pretreated with 0.5 M NaOH at 80°C for 75 minutes at a solid-to-liquid ratio of 1:8 (w/v) according to a previously published procedure [21]. The solid residues were collected by filtration, washed with water three times to reach a neutral pH, dried at 105°C to a constant weight, and stored in a desiccate.

### Enzymatic hydrolysis of alkaline-pretreated corn stover

The alkaline-pretreated corn stover was loaded at 10% (w/v) solid loading in 50 mM citrate (pH 4.8) and hydrolyzed at 50°C for 48 hours in the presence of different amounts of cellulase, β-glucosidase and xylanase. Recovered enzymes were used under identical conditions, but different amounts of cellulase and xylanase were supplemented when necessary.

### Separated hydrolysis and enhanced lipid production process

Corn stover hydrolysates were prepared in 50 mM potassium phosphate (pH 4.8) under identical conditions for enzymatic hydrolysis. *C. curvatus* ATCC 20509 pre-cultures were grown in YPD liquid medium for 24 hours at 30°C and 200 rpm. Cells were collected by centrifugation and used as inocula. One unit optical absorbance at 600 nm (OD_600_) for such inocula was equivalent to a cell density of 0.36 g of cell dry weight (CDW) per liter.

Experiments were performed in 500 mL conical flasks with 50 mL of corn stover hydrolysates (pH 5.5) supplemented with 2.0 g/L Tween 80 and 50 mg/L ampicillin (Dingguo Biotechnology Co. Ltd., Beijing, China). Cultures were initiated by resuspending inocula cells equivalent to 0.36 g of CDW in the corn stover hydrolysates, and then held for 71 hours at 30°C and 200 rpm.

### Simultaneous saccharification and lipid production process

Experiments were carried out in 500 mL conical flasks in 50 mM potassium phosphate (pH 5.2), or a nutrient solution (pH 5.2) with 5.0 g of corn stover at 10% (w/v) solid loading supplemented with 2.0 g/L Tween 80, 50 mg/L ampicillin, 10 FPU/g cellulase and 80 U/g xylanase. Cultures were initiated by 5.0 mL of the pre-culture (corresponding to 45 mg CDW cells), and held for 72 hours at 30°C and 200 rpm. The nutrient solution was composed of yeast extract 1.0 g/L, KH_2_PO_4_ 2.7 g/L, Na_2_HPO_4_ · 7H_2_O 2.4 g/L, MgSO_4_ · 7H_2_O 0.2 g/L, ethylenediaminetetraacetic acid 0.1 g/L, Tween 80 2.0 g/L, 1% (v/v) trace element solution and various amounts of (NH_4_)_2_SO_4_. The composition of the trace element solution contained (g/L): CaCl_2_ · 2H_2_O 4.0, FeSO_4_ · 7H_2_O 0.55, citric acid · H_2_O 0.52, ZnSO_4_ · 7H_2_O 0.10, MnSO_4_ · H_2_O 0.076 and H_2_SO_4_ 0.18 [22].

### Simultaneous saccharification and enhanced lipid production process

Experiments were performed in 500 mL conical flasks in 50 mM phosphate buffer (pH 5.2) with 5.0 g of corn stover at 10% (w/v) solid loading supplemented with 2.0 g/L Tween 80, 50 mg/L ampicillin and various amounts of cellulolytic enzymes. Cultures were initiated by inocula cells equivalent to 0.36 g of CDW, and then held for 72 hours at 30°C and 200 rpm.

### Simultaneous saccharification and enhanced lipid production process with enzyme recycling

Enzyme recycling experiments were done using broth samples of the SSELP process supplemented with 10 FPU/g cellulase and 80 U/g xylanase. The broths were centrifuged at 20,000 g to separate the solids and the supernatants. The solids were mixed thoroughly with about 9 mL of 50 mM phosphate (pH 5.2), centrifuged, and the supernatants were combined to a total volume of 50 mL.

The second round SSELP process was done using the spent supernatants with no extra enzyme loading under otherwise identical conditions.

All experiments were done in triplicate and data were presented as mean value ± standard deviation.

### Analytical methods

Glucose was determined using an SBA-50B glucose analyzer (Shandong Academy of Sciences, Jinan, China). Total reducing sugars (TRS) were quantified according to the 2,4-dinitrosalicylate method with glucose as standard [23]. Sugar mixtures were analyzed by ion chromatography (IC) on the Dionex ICS2500 system with a CarboPac PA10 analytical column and an ED50 electrochemical detector (Dionex, Sunnyvale, USA). The column was washed with an isocratic elution of NaOH at a speed of 1 mL/min at 30°C.

Structural carbohydrates and lignin content of corn stover were analyzed according to the procedures of the National Renewable Energy Laboratory [24].

Total lipids of cells prepared by the SHELP process were extracted according to the chloroform/methanol method [13]. Total lipids of the SSLP and SSELP processes were obtained by extraction according to the chloroform/methanol method followed by a petroleum-ether partition step. Briefly, total mass containing cells and corn stover residues was harvested by centrifugation, washed twice with distilled water and determined gravimetrically after drying at 105°C for 24 hours. Crude lipids were extracted from dried total mass, dissolved again with petroleum ether and filtrated to recover the petroleum-ether solution. The solvents were removed by rotary evaporation, and the residue was dried at 105°C for 24 hours to give the total lipids [14]. Lipid content was expressed as gram total lipids per gram CDW. The lipid coefficient was defined as milligram total lipids produced per gram corn stover supplied, as has been adopted by others in this field [3].

Lipid fractionation was performed according to a published method, with minor modifications [25]. About 100 mg of the lipid sample was loaded on a silica gel column (Waters Sep-Pak™ Vac 6 cc Slilca-1 g (Waters Corporation, Milford, USA), eluted by 100 mL of 1,2-dichloroethane, 70 mL of 1,2-dichloroethane/acetone (1:10, v/v) and 35 mL of methanol in sequence. The solvents from each fraction were evaporated in a vacuum, and the weight of the residual lipid was determined. The fractions, in the order of elution, were neutral lipids (N), glycolipids plus sphingolipids (G + S) and phospholipids (P). The fatty acid compositional profiles of lipid samples were determined on a 7890 F gas chromatography instrument (Techcomp Scientific Instrument Co. Ltd., Shanghai, China) after transmethylation according to a published procedure [26].

### Statistical analysis

An analysis of variance (ANOVA) and multiple comparisons were performed to identify the significant differences of the experimental data. Tukey’s *post hoc* test was conducted to compute significant differences among the experimental data of various culture modes. Bonferroni’s *post hoc* test was adopted to calculate multiple comparisons of experimental data obtained under different hydrolysis or culture conditions. A *P* value of less than 0.05 was considered statistically significant.

## Results and discussion

### Separated hydrolysis and enhanced lipid production process

The chemical composition of raw corn stover samples used in this study contained 37.9% glucan, 20.1% xylan, 2.3% arabinan, 0.9% galactan, 0.3% mannan and 20.8% lignin. The alkaline pretreatment was efficient at removing lignin by disrupting the ester bonds cross-linking lignin and hemicellulose, as well as enhancing the enzyme accessibility of cellulose [21]. In a typical alkaline pretreatment process, the regenerated corn stover was received in a yield of 61.6%. The chemical composition of the regenerated sample contained 56.2% glucan, 16.5% xylan, 3.1% arabinan, 0.5% galactan, 0.5% mannan and 8.2% lignin, indicating that more than 75% of lignin was removed.

**Figure 1:**
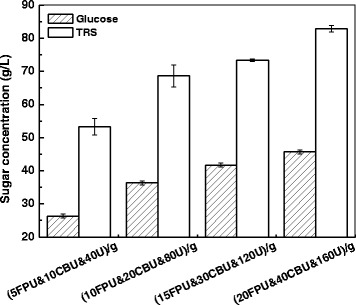
**Effects of enzyme dosage on corn stover hydrolysis.** CBU, cellobiase unit; FPU, filter paper unit; TRS, total reducing sugars.

To ensure complete hydrolysis of the regenerated corn stover, different amounts of cellulolytic enzymes were used, and final concentrations of glucose and TRS after 48 hours are shown in Figure 1. It was clear that higher enzyme loadings afforded more products. When cellulase, β-glucosidase and xylanase were loaded at 5 FPU, 10 CBU and 40 U/g regenerated corn stover, glucose and TRS were only 26.3 g/L and 53.3 g/L, respectively. When enzymes loadings were all doubled, glucose and TRS increased significantly (*P* <0.05) to 36.3 g/L and 68.6 g/L, respectively. When cellulase, β-glucosidase and xylanase increased further to 20 FPU, 40 CBU and 160 U/g, glucose and TRS reached 45.7 g/L and 82.8 g/L, respectively. As the initial corn stover concentration was 10% (w/v), the formation of 82.8 g/L TRS suggested that over 95% polymeric carbohydrates were hydrolyzed to soluble reducing sugars. Therefore, the combination of 20 FPU/g cellulase, 40 CBU/g β-glucosidase and 160 U/g xylanase was sufficient to hydrolyze the regenerated corn stover sample.

The hydrolysates were prepared with the highest enzyme loadings, separated by centrifugation, and used to culture *C. curvatus* according to the SHELP process. After 71 hours, glucose concentration was below the limit of detection and residual TRS was only 2.8 g/L in the culture broth. Cell mass, lipid content, lipid coefficient and lipid productivity were 27.7 g/L, 43.6%, 155.9 mg/g initial TRS and 3.75 g/L/d, respectively. The lipid coefficient was equivalent to 115.6 mg/g alkaline-pretreated corn stover.

### Simultaneous saccharification and lipid production process

As glucose and cellobiose are strong inhibitors for cellulase, hydrolysis of biomass usually requires relatively large amounts of enzymes due to markedly slowing down the reaction over time [27]. The SSLP process may be applied to overcome product inhibition and reduce enzyme usage for the conversion of cellulosic biomass into lipids. In fact, cellulose was effectively converted into lipids by *C. curvatus* in the presence of cellulase, but without the addition of exogenous β-glucosidase [13].

**Figure 2:**
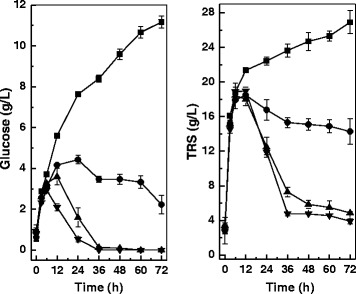
**Sugar evolution profiles of the** **simultaneous saccharification and lipid production process by** ***Cryptococcus curvatus*** **in different solutions.** Square, potassium phosphate buffer; circle, nutrient solution plus 0.1 g/L (NH_4_)_2_SO_4_; triangle, nutrient solution plus 0.5 g/L (NH_4_)_2_SO_4_; inverted triangle, nutrient solution plus 1.0 g/L (NH_4_)_2_SO_4_. TRS, total reducing sugars.

When alkaline-pretreated corn stover was directly used according to the SSLP process with *C. curvatus* in potassium phosphate buffer, lipid titre, lipid coefficient and lipid productivity were only 6.2 g/L, 67.2 mg/g and 2.10 g/L/d, respectively (Table 1, Entry 1). Glucose and TRS accumulated up to 11.2 g/L and 26.9 g/L, respectively, when the culture was terminated at 72 hours, indicating substrate consumption was rate-limiting (Figure 2). This was likely due to poor cell growth under a nutrient-famine environment. To improve cell growth and lipid production, experiments were done in the nutrient solution in the presence of different amounts of (NH_4_)_2_SO_4_. When 0.1 g/L (NH_4_)_2_SO_4_ was applied, lipid titre, lipid coefficient and lipid productivity increased significantly (*P* <0.05) to 10.0 g/L, 108.0 mg/g and 3.37 g/L/d, respectively (Table 1, Entry 2). Residual glucose and TRS dropped to 2.2 g/L and 14.3 g/L, respectively. Lipid titre, lipid coefficient and lipid productivity increased further to 11.9 g/L, 129.4 mg/g and 4.03 g/L/d, respectively, when 0.5 g/L (NH_4_)_2_SO_4_ was used (Table 1, Entry 3). It was found that glucose concentration was below the limit of detection after 36 hours, indicating that glucose formation by enzymatic hydrolysis was slower than glucose consumption due to the accumulation of *C. curvatus* cells. However, slightly lower lipid titre and lipid coefficient were observed when 1.0 g/L (NH_4_)_2_SO_4_ was added (Table 1, Entry 4), but these differences appeared not to be significant (*P* >0.05). This was in accordance with the general trends that oleaginous yeasts favor cell proliferation rather than lipid biosynthesis in the presence of excess nutrients, especially the nitrogen source [28].

**Table 1:** **Results of the** s**imultaneous saccharification and lipid production process in the presence of extra nutrients**

**Entry**	**(NH**_**4**_ **)**_**2**_ **SO**_**4**_ **(g/L)**	**Residual total reducing sugars (g/L)**	**Lipid (g/L)**	**Lipid coefficient (mg/g)**	**Lipid productivity (g/L/d)**
1	-	26.9 ± 1.3	6.2 ± 0.8	67.2 ± 8.9	2.10 ± 0.28
2	0.1	14.3 ± 1.5	10.0 ± 0.6	108.0 ± 6.0	3.37 ± 0.19
3	0.5	4.9 ± 0.1	11.9 ± 0.2	129.4 ± 2.3	4.03 ± 0.07
4	1.0	3.9 ± 0.3	11.1 ± 0.2	120.3 ± 2.3	3.75 ± 0.07

It should be emphasized that no β-glucosidase was used, and cellulase and xylanase were loaded only 10 FPU and 80 U, respectively, per gram of alkaline-pretreated corn stover in the SSLP process, which were dramatically less than those in the SHELP process. Yet, lipid coefficient was increased by 12% compared to that of the SHELP process. A similar example with acid-pretreated corn stover using *Trichosporon cutaneum* as the lipid producer was recently reported [14], but lipid coefficient and lipid productivity were 32.3 mg/g and 0.86 g/L/d, respectively, which were much lower than our results.

### Simultaneous saccharification and enhanced lipid production process

**Figure 3:**
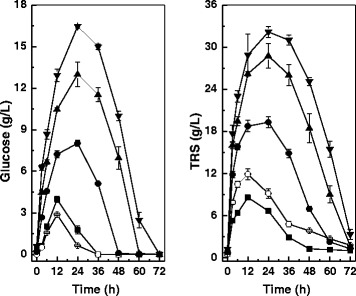
**Sugar evolution profiles of the s** **imultaneous saccharification and enhanced lipid production process by** ***Cryptococcus curvatus*** **in the presence of different cellulolytic enzymes.** Enzyme amounts for cellulase in FPU, β-glucosidase in CBU and xylanase in U, respectively. square, 5 FPU, 10 CBU and 40 U/g; empty circle, 10 FPU and 80 U/g; filled circle, 10 FPU, 20 CBU and 80 U/g; triangle, 15 FPU, 30 CBU and 120 U/g; inverted triangle, 20 FPU, 40 CBU and 160 U/g. CBU, cellobiase unit, FPU, filter paper unit; TRS, total reducing sugars.

To further improve lipid production efficiency, we tested the SSELP process, and the results are shown in Table 2. The evolution of glucose and TRS in the culture broth shared similar trends (Figure 3). There was an apparent sugar formation burst, followed by a short plateau and then a gradual decline. Higher enzyme loadings afforded longer plateau time and higher sugar concentrations. When enzyme loadings were relatively low, glucose was below the limit of detection for a longer culture time period. When cellulase, β-glucosidase and xylanase loadings were 5 FPU/g, 10 CBU/g and 40 U/g, the concentrations of glucose and TRS after 12 hours were 4.0 g/L and 8.6 g/L, respectively. Glucose was below the limit of detection and TRS was 2.9 g/L after 36 hours, suggesting that the sugars became limiting for yeast utilization. TRS was only 1.0 g/L at the end of the culture, indicating that *C. curvatus* had an exceptional capacity of assimilating different sugar components generated by enzymatic hydrolysis. Lipid titre, lipid coefficient and lipid productivity were 13.2 g/L, 137.9 mg/g and 4.06 g/L/d, respectively (Table 2, Entry 1). When cellulase, β-glucosidase and xylanase were all doubled, the concentrations of glucose and TRS were 8.0 g/L and 19.3 g/L, respectively. At the end of the culture, no glucose was observed and TRS was only 1.3 g/L. Lipid titre, lipid coefficient and lipid productivity increased to 14.4 g/L, 151.8 mg/g and 4.47 g/L/d, respectively (Table 2, Entry 3). The concurrent decrease in total mass and increase in lipid titre suggested that more sugars were released from corn stover and converted into lipids.

**Table 2:** **Results of the** s**imultaneous saccharification and enhanced lipid production process in the presence of different amounts of cellulolytic enzymes**

**Entry**	Enzymes loading (/g corn stover)*	**Total mass (g/L)**	**Residual TRS (g/L)**	**Lipid (g/L)**	**Lipid coefficient (mg/g)**	**Lipid productivity (g/L/d)**
1	5 FPU, 10 CBU and 40 U	57.2 ± 0.9	1.0 ± 0.1	13.2 ± 0.3	137.9 ± 3.6	4.06 ± 0.11
2	10 FPU and 80 U	47.3 ± 0.4	1.8 ± 0.0	15.1 ± 0.1	159.4 ± 1.3	4.69 ± 0.04
3	10 FPU, 20 CBU and 80 U	48.5 ± 0.5	1.3 ± 0.0	14.4 ± 0.2	151.8 ± 2.0	4.47 ± 0.06
4	15 FPU, 30 CBU and 120 U	49.3 ± 0.4	1.6 ± 0.2	14.1 ± 0.6	148.1 ± 6.8	4.36 ± 0.20
**5**	20 FPU, 40 CBU and 160 U	49.9 ± 0.5	3.3 ± 0.7	13.4 ± 0.7	140.1 ± 8.0	4.12 ± 0.23

*Enzyme activities are indicated for cellulase in FPU, β-glucosidase in CBU and xylanase in U, respectively. CBU, cellobiase unit; FPU, filter paper unit; TRS, total reducing sugars.

When more enzymes were loaded, there was no further improvement for lipid production. In contrast, lipid titre and lipid coefficient both dropped slightly and total mass increased (Table 2, Entry 4 and 5), but Bonferroni’s test suggested that these differences were not significant (*P* >0.05). For the experiment with cellulase, β-glucosidase and xylanase loadings at 20 FPU/g, 40 CBU/g and 160 U/g, respectively, glucose and TRS after 24 hours were 16.5 g/L and 32.2 g/L, respectively. It was obvious that sugars were released faster than assimilation by *C. curvatus* cells. The fact that lipid coefficient dropped slightly at high enzyme loadings might be due to the concurrent incorporation of excess nutrients such as the nitrogen source in the broth. In separate experiments using glucose-based nitrogen limited media for lipid production [26], we also found that the addition of cellulase and β-glucosidase led to better cell growth but lower lipid contents (data not shown).

It was interesting to note that the best results were obtained in the absence of exogenous β-glucosidase (Table 2, Entry 2). The maximal glucose concentration was only 2.9 g/L, and TRS was 1.8 g/L at the end of culture. Lipid titre, lipid coefficient and lipid productivity reached 15.1 g/L, 159.4 mg/g alkaline-pretreated corn stover and 4.69 g/L/h, respectively. Lipid coefficient was improved by 37.9% and 23.2% compared to that of the SHELP and SSLP processes, respectively. Such good results might be due to optimal nutrient limitation because of less enzyme loadings, and to well-matched rates between enzymatic hydrolysis and lipid production.

### Enzyme recovery

To test enzyme stability during the culture process, enzyme samples were first incubated under identical conditions for 71 hours, and then loaded with 10% (w/v) alkaline-pretreated corn stover samples for hydrolysis. Glucose and TRS reached 42.0 g/L and 73.7 g/L after 36 hours, while the corresponding fresh enzymes gave glucose and TRS at 43.7 g/L and 76.9 g/L, respectively. These results suggested that the culture process had minor detrimental effects on enzyme stability. When the corn stover samples were treated by the spent culture supernatants of the SSELP process initially loaded with 10 FPU/g cellulase and 80 U/g xylanase, concentrations of glucose and TRS after 48 hours were 9.3 g/L and 31.2 g/L, respectively. However, hydrolysis with identical amounts of fresh enzymes gave glucose and TRS at 23.3 g/L and 55.5 g/L, respectively. These results indicate that substantial enzyme activities were recovered. To further demonstrate the possibility of enzyme recovery, the spent supernatants were loaded with 10% (w/v) alkaline-pretreated corn stover and *C. curvatus* cells equivalent to 0.36 g of CDW for the second SSELP run. Lipid titre and lipid coefficient were 4.9 g/L and 44.1 mg/g, which were 32.7% and 27.9%, respectively, of those from the fresh enzymes used in the first run. It should be noted that alkaline-pretreated corn stover contained about 8.2% lignin, which might entrap some enzymes and reduce enzyme recovery efficiency. Interestingly, desorption strategies were known to improve enzyme recovery from the biomass hydrolysis mixtures [29]. Therefore, it is expected that more enzymes can be recycled from the SSELP process upon further optimization.

### Comparison between processes for lipid production from corn stover

Microbial lipid samples from different processes were analyzed and the results are shown in Table 3. It was found that fatty acids with 16 and 18 carbon atoms were the majority, and the fatty acid compositional profiles were similar to those of vegetable oils, suggesting that microbial lipids were suitable for biodiesel production [30]. Lipid samples from the SSLP and SSELP processes contained neutral lipids around 95%, which were apparently higher than that of the SHELP process. The difference was likely caused by an additional petroleum-ether extraction step used to extract lipids in the SSLP and SSELP processes, which removed major polar lipids including glycolipids, sphingolipids and phospholipids because of their poor solubility in petroleum ethers. Also, higher contents of unsaturated fatty acids, including oleic acid and linoleic acid, were found in lipid samples from the SSLP and SSELP processes. However, direct comparison on fatty acid compositional profiles were not appropriate because these samples were from different cultivation and extraction processes.

**Table 3:** **Lipid fractions and fatty acid compositions of lipid samples produced from corn stover by** ***Cryptococcus curvatus*** **under different culture modes**

**Culture mode**	**Lipid fractions (% w/w)**			**Relative fatty acid content (% w/w)**					
	**N**	**G + S**	**P**	**Myristic acid**	**Palmitic acid**	**Palmitoleic acid**	**Stearic acid**	**Oleic acid**	**Linoleic acid**
SHELP	81.9 ± 1.4	14.8 ± 1.2	3.3 ± 0.2	1.2 ± 0.1	29.4 ± 0.7	0.6 ± 0.2	18.4 ± 0.5	44.1 ± 2.3	1.5 ± 0.3
SSLP	94.3 ± 0.7	3.6 ± 0.5	2.1 ± 0.2	0.7 ± 0.1	27.2 ± 0.9	0.7 ± 0.2	11.2 ± 0.7	52.5 ± 1.7	6.1 ± 0.2
SSELP	96.1 ± 0.5	2.3 ± 0.6	1.6 ± 0.2	0.6 ± 0.3	25.3 ± 0.3	0.6 ± 0.1	14.6 ± 0.1	51.1 ± 0.4	5.9 ± 0.6

N, neutral lipids; G + S, glycolipids plus sphingolipids; P, phospholipids; SHELP, separated hydrolysis and enhanced lipid production; SSELP, simultaneous saccharification and enhanced lipid production; SSLP, simultaneous saccharification and lipid production.

**Figure 4:**
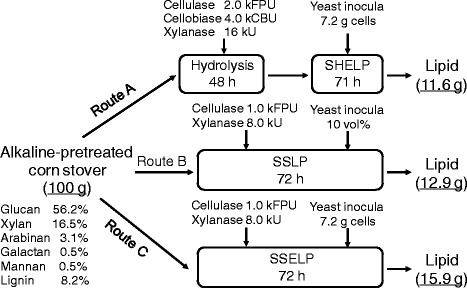
**Flow chart indicating mass balance of lipid production from corn stover by** ***Cryptococcus curvatus*** **under different culture modes.** CBU, cellobiase unit; FPU, filter paper unit; SHELP, separated hydrolysis and enhanced lipid production; SSELP, simultaneous saccharification and enhanced lipid production; SSLP, simultaneous saccharification and lipid production.

Results of three processes for lipid production from alkaline-pretreated corn stover by *C. curvatus* are summarized in Figure 4. A one-way ANOVA followed by Tukey’s *post hoc* test demonstrated that lipid coefficients increased significantly among different processes following the order of SHELP, SSLP and SSELP (*P* <0.05). The highest lipid coefficient of 15.9 g/100 g alkaline-pretreated corn stover, equal to 9.8 g/100 g raw corn stover, was obtained for the SSELP process, when 1.0 kFPU cellulase and 8.0 kU xylanase were used (Route C). It should be pointed out that the lipids were slightly underestimated for the SSLP and SSELP processes because polar lipids were partially removed by the petroleum-ether partition step. Therefore, lipid coefficient values of the SSLP and SSELP processes were conservative. Compared with the SHELP process, both cellulase and xylanase loadings were reduced by 50% and no β-glucosidase was used for the SSELP process. Lipid coefficient of 12.9 g/100 g alkaline-pretreated corn stover, corresponding to 7.9 g/100 g raw corn stover, was observed for the SSLP process. For the SHELP process, lipid coefficient was only 11.6 g/100 g alkaline-pretreated corn stover (Route A). The SHELP process gave the lowest lipid coefficient, likely due to TRS loss in the hydrolysates recovery step and the presence of more nutrients originating from higher enzyme loadings. Because β-glucosidase, additional equipment and longer time were required, the SHELP process was apparently less cost-effective than the other two processes.

Recently, lignocellulosic materials have been explored as feedstocks for microbial lipid production by different oleaginous microbes with different processes [3,14,31-34]. For example, enzymatic hydrolysis and lipid production by the yeast *Trichosporon cutaneum* were integrated in a 50 L stirred-tank bioreactor, where lipid titre, lipid coefficient and lipid productivity were only 3.23 g/L, 32.3 mg/g corn stover and 0.86 g/L/d, respectively [14]. Lipid production by *Microsphaeropsis sp.* was carried out using steam-exploded wheat straw under solid-state culture conditions, and lipid coefficient was about 80 mg/g [33]. As shown in Table 4, the SSELP process had higher lipid coefficient and lipid productivity over those of previous results. This was because the SSELP process had the advantage of a two-stage lipid production [35] together with those of simultaneous saccharification and fermentation process being widely explored in biorefinery research [11,12]. As lipids are intracellular products, the SSELP process also provided an opportunity to recover and reuse cellulolytic enzymes more conveniently, which could further reduce enzyme consumption and wastewater discharge.

**Table 4:** **Lipid coefficients of lipid production from lignocellulosic materials by different oleaginous microorganisms**

**Oleaginous microorganism**	**Carbon source**	**Cultivation mode**	**Lipid coefficient (mg/g)**	**Lipid productivity (g/L/d)**	**Reference**
*Cryptococcus curvatus*	Sorghum bagasse	Batch	84.7	0.88	[31]
*Rhodosporidium toruloides* Y4	Corn stover	Batch	71.0	0.84	[32]
*Mortierella isabellina*	Corn stover	Co-hydrolysis and fermentation	62.8	0.87	[3]
*Microsphaeropsis* sp.	Wheat straw + bran	Solid-state fermentation	80.0	-	[33]
*Aspergillus oryzae* A-4	Wheat straw + bran	Solid-state fermentation	62.9	-	[34]
*Trichosporon cutaneum*	Corn stover	Simultaneous saccharification and fermentation	32.3	0.86	[14]
*Cryptococcus curvatus*	Corn stover	Simultaneous saccharification and enhanced lipid production	98.2	4.69	This study

## Conclusions

The current study compared three different processes for microbial lipid production from alkaline-pretreated corn stover by *C. curvatus*. It was found that the SSELP process was the most promising one, which consumed less enzymes and afforded higher lipid coefficient. Moreover, enzymes could be partially recovered by recycling the spent culture supernatants, which could further reduce enzyme dosage and wastewater discharge. These results provide valuable information for cost-effective lipid production from lignocelluloses.

## Abbreviations

CBU: Cellobiase unit
CDW: Cell dry weight
FPU: Filter paper unit
G: Glycolipids
IC: Ion chromatography
N: Neutral lipids
P: Phospholipids
S: Sphingolipids
SHELP: Separated hydrolysis and enhanced lipid production
SSELP: Simultaneous saccharification and enhanced lipid production
SSLP: Simultaneous saccharification and lipid production
TRS: Total reducing sugar
YPD: Yeast peptone dextrose
